# Supplementary material for: Double lives: transfer of fungal endophytes from leaves to woody substrates
Source: PeerJ. 2020 Aug 28;8:e9341. doi: 10.7717/peerj.9341 (PMC7457945; doi:10.7717/peerj.9341)
Supplement: Supplemental Information 1 [file peerj-08-9341-s001.docx]

**Table S1: Identifications of representative isolates from each morphotype**

| **Morpho-type** | **GenBank Accession** | **Species Identification** | **UNITE SH Identifier** | **Notes** |
| --- | --- | --- | --- | --- |
| 1 | MN421851 | *Phlebia radiata* Fr. | SH1599717.08FU_UDB015454_refs | |
| 2 | MN421852 | *Neopestalotiopsis foedans* (Sacc. & Ellis) Maharachch., K.D. Hyde & Crous | SH1552672.08FU_JX398987_refs | |
| 3 | MN421853 | *Trichoderma evansii* Samuels | SH1568468.08FU_EU883568_refs | |
| 4 | MN421854 | *Lophiostoma sp.* | SH1613292.08FU_KC978009_reps | |
| 5 | MN421855 | *Neopestalotiopsis foedans* (Sacc. & Ellis) Maharachch., K.D. Hyde & Crous | SH1552672.08FU_JX398987_refs | |
| 6 | MN421858 | *Phanerochaete cumulodentata* (Nikolajeva) E. Parmasto*.* | SH1573643.08FU_JX623930_reps | |
| 7 | MN421859 | *Diaporthe sp.* | SH1908675.08FU_JX436799_reps | |
| 8 | MN421860 | *Neopestalotiopsis foedans* (Sacc. & Ellis) Maharachch., K.D. Hyde & Crous | SH1552672.08FU_JX398987_refs | |
| 9 | — | — | — | Bad sequence; morphologically identified as a *Trichoderma spp.* |
| 10 | MN421861 | *Neopestalotiopsis foedans* (Sacc. & Ellis) Maharachch., K.D. Hyde & Crous | SH2251709.08FU_JX398983_refs | |
| 11 | MN421862 | *Xylaria berteri* (Mont.) Cooke ex J.D. Rogers & Y.M. Ju | SH1541120.08FU_GU324749_reps | Sporocarps in RLC collections: RLC1558 (GenBank accession: KP133344) |
| 12 | MN421864 | *Byssochlamys spectabilis* (Udagawa & Shoji Suzuki) Houbraken & Samson | SH1530695.08FU_MG654695_reps | |
| 13 | — | — | — | Bad sequence; morphologically identified as a *Paecilomyces spp.* |
| 14 | MN421865 | *Curvularia affinis* Boedijn | SH1688433.08FU_KM230400_refs | |
| 15 | MN421866 | *Colletotrichum kahawae* J.M. Waller & Bridge | SH2219005.08FU_JX010230_refs | |
| 16 | MN421867 | Dothideomycetes | SH1579758.08FU_EU781672_reps | |
| 17 | MN421868 | *Curvularia affinis* Boedijn | SH1688433.08FU_KM230400_refs | |
| 18 | MN421869 | *Kalmusia variispora* (Verkley, Göker & Stielow) Ariyawansa & K.D. Hyde | SH1387397.08FU_JX496030_reps | |
| 19 | MN421870 | *Cladophialophora sp.* | SH1562574.08FU_LC192127_reps | |
| 20 | MN421871 | *Fusarium oxysporum* Schltdl. | SH2456720.08FU_DQ452451_refs_singleton | |
| 21 | MN421863 | *Alloconiothyrium aptrootii* Verkley, Göker & Stielow | SH1525467.08FU_JX496121_refs | |
| 22 | MN421872 | *Byssochlamys spectabilis* (Udagawa & Shoji Suzuki) Houbraken & Samson | SH1530695.08FU_MG654695_reps | |
| 23 | MN421873 | *Aspergillus fresenii* Subram. | SH1530483.08FU_KJ775477_reps | |
| 24 | MN421874 | *Penicillium citrinum* Thom | SH1529986.08FU_AF033422_refs | |
| 25 | MN421875 | *Neopestalotiopsis foedans* (Sacc. & Ellis) Maharachch., K.D. Hyde & Crous | SH1552672.08FU_JX398987_refs | |
| 26 | MN421876 | *Peniophora pithya* (Pers.) J. Erikss | SH1646425.08FU_KM265849_reps | |
| 27 | MN421877 | *Peniophora pithya* (Pers.) J. Erikss | SH1646425.08FU_KM265849_reps | |
| 28 | MN421878 | *Alloconiothyrium aptrootii* Verkley, Göker & Stielow | SH1525467.08FU_JX496121_refs | |
| 29 | MN421879 | Nectriaceae | SH2228369.08FU_KU204663_reps | |
| 30 | MN421880 | *Neopestalotiopsis foedans* (Sacc. & Ellis) Maharachch., K.D. Hyde & Crous | SH1552672.08FU_JX398987_refs | |
| 31 | MN421881 | *Curvularia affinis* Boedijn | SH1688433.08FU_KM230400_refs | |
| 32 | MN421882 | *Byssochlamys spectabilis* (Udagawa & Shoji Suzuki) Houbraken & Samson | SH1530695.08FU_MG654695_reps | |
| 33 | MN421883 | *Phlebiopsis gigantea* (Fr.) Jülich | SH1573645.08FU_AM084460_refs | |
| 34 | MN421884 | *Pestalotiopsis adusta* (Ellis & Everh.) Steyaert | SH2289918.08FU_JX399006_refs | |
| 35 | — | — | — | Bad extraction; excluded from further analysis |
| 36 | MN421885 | *Fusarium oxysporum* Schltdl. | SH2456720.08FU_DQ452451_refs_singleton | |
| 37 | — | — | — | Bad sequence; morphologically identified as a *Neopestalotiopsis spp.* |
| 38 | MN421886 | *Phlebia radiata* Fr. | SH1599717.08FU_UDB015454_refs | |
| 39 | MN421887 | *Peniophora pithya* (Pers.) J. Erikss | SH1646425.08FU_KM265849_reps | |
| 40 | MN421888 | *Coprinellus sp.* | SH1508476.08FU_KM265600_reps | Sporocarps in RLC collections: RLC159, RLC372, RLC705, RLC788 |
| 41 | MN421889 | *Kalmusia variispora* (Verkley, Göker & Stielow) Ariyawansa & K.D. Hyde | SH1387397.08FU_JX496030_reps | |
| 42 | — | — | — | Bad extraction; excluded from further analysis |
| 43 | MN421890 | *Muscodor sp.* | SH1866862.08FU_MG309793_reps | |
| 44 | MN421891 | *Diaporthe sp.* | SH1908675.08FU_JX436799_reps | |
| 45 | MN421892 | *Phanerochaete cumulodentata* (Nikolajeva) E. Parmasto | SH1573643.08FU_KP994359_reps | |
| 46 | MN421893 | *Peniophora pithya* (Pers.) J. Erikss | SH1646425.08FU_KM265849_reps | |
| 47 | MN421894 | *Kalmusia variispora* (Verkley, Göker & Stielow) Ariyawansa & K.D. Hyde | SH1387397.08FU_JX496030_reps | |
| 48 | MN421895 | *—* | SH1930846.08FU_KF412647_refs | Sequenced cross-contaminant; excluded from further analysis |
| 49 | MN421896 | *Phlebiopsis gigantea* (Fr.) Jülich | SH1573645.08FU_AM084460_refs | |
| 50 | MN421897 | *Neopestalotiopsis foedans* (Sacc. & Ellis) Maharachch., K.D. Hyde & Crous | SH1552672.08FU_JX398987_refs | |
| 51 | MN421898 | *Neopestalotiopsis foedans* (Sacc. & Ellis) Maharachch., K.D. Hyde & Crous | SH1552672.08FU_JX398987_refs | |
| 52 | MN421899 | *Peniophora pithya* (Pers.) J. Erikss | SH1646425.08FU_KM265849_reps | |
| 53 | MN421900 | *Phlebia tremellosa* Schrad. Nakasone & Burds. | SH1576304.08FU_UDB011413_refs | |
| 54 | MN421901 | *Peniophora pithya* (Pers.) J. Erikss | SH1646425.08FU_KM265849_reps | |
| 55 | MN421902 | *Neopestalotiopsis foedans* (Sacc. & Ellis) Maharachch., K.D. Hyde & Crous | SH1552672.08FU_JX398987_refs | |
| 56 | MN421903 | *Curvularia affinis* Boedijn | SH1688433.08FU_KM230400_refs | |
| 57 | MN421904 | *Hypoxylon trugodes* Berk. & Broome | SH1515887.08FU_JQ009319_reps | |
| 58 | MN421905 | *Mycoacia sp.* | SH1564277.08FU_HM583818_refs | |
| 59 | MN421906 | *Fusarium oxysporum* Schltdl. | SH2456720.08FU_DQ452451_refs_singleton | |
| 60 | MN421907 | *Aspergillus subramanianii* Visagie, Frisvad & Samson | SH1530325.08FU_KT959281_reps | |
| 61 | MN421908 | *Byssochlamys spectabilis* (Udagawa & Shoji Suzuki) Houbraken & Samson | SH1530695.08FU_MG654695_reps | |
| 62 | MN421909 | *Alloconiothyrium aptrootii* Verkley, Göker & Stielow | SH1525467.08FU_JX496121_refs | |
| 63 | MN421910 | *Neopestalotiopsis foedans* (Sacc. & Ellis) Maharachch., K.D. Hyde & Crous | SH1552672.08FU_JX398987_refs | |
| 64 | — | — | — | Bad extraction; excluded from further analysis |
