# Supplementary material for: Double lives: transfer of fungal endophytes from leaves to woody substrates
Source: PeerJ. 2020 Aug 28;8:e9341. doi: 10.7717/peerj.9341 (PMC7457945; doi:10.7717/peerj.9341)
Supplement: Supplemental Information 2 [file peerj-08-9341-s002.docx]

**Table S2: Taxonomy of cultured viaphyte genera from this study**

| **Phylum** | **Class** | **Order** | **Family** | **Genus** | **Total Isolates** |
| --- | --- | --- | --- | --- | --- |
| Ascomycota | Eurotiomycestes | Eurotiales | Trichomaceae | *Penicillium* | 179 |
| Ascomycota | Sordariomycetes | Hypocreales | Hypoceaceae | *Trichoderma* | 89 |
| Ascomycota | Eurotiomycestes | Eurotiales | Trichocomaceae | *Byssochlamys* | 75 |
| Ascomycota | Sordariomycetes | Amphisphaeriales | Pestalotiopsidaceae | *Neopestalotiopsis* | 44 |
| Basidiomycota | Agaricomycetes | Polyporales | Phanerochaetaceae | *Phanerochaete* | 14 |
| Ascomycota | Pyrenomycetes | Diaporthales | Diaporthaceae | *Diaporthe* | 10 |
| Ascomycota | Sordariomycetes | Hypocreales | Nectriaceae | *Fusarium* | 9 |
| Ascomycota | Eurotiomycestes | Eurotiales | Aspergillaceae | *Aspergillus* | 6 |
| Ascomycota | Dothideomycetes | Pleosporales | Pleosporaceae | *Curvularia* | 6 |
| Basidiomycota | Agaricomycetes | Russulales | Peniophoraceae | *Peniophora* | 6 |
| Ascomycota | Dothideomycetes | Pleosporales | Didymosphaeriaceaee | *Alloconiothyrium* | 5 |
| Ascomycota | Dothideomycetes | Pleosporales | Didymosphaeriaceaee | *Kalmusia* | 5 |
| Basidiomycota | Agaricomycetes | Polyporales | Meruliaceae | *Phlebia* | 4 |
| Ascomycota | Eurotiomycestes | Chaetothyriales | Herpotrichiellaceae | *Cladophialophora* | 3 |
| Ascomycota | Sordariomycetes | Xylariales | Xylariaceae | *Xylaria* | 3 |
| Ascomycota | Sordariomycetes | Glomerellales | Glomerellaceae | *Colletotrichum* | 2 |
| Ascomycota | Dothideomycetes | Pleosporales | Lophiostomataceae | *Lophiostoma* | 2 |
| Ascomycota | Dothideomycetes | — | — | — | 2 |
| Basidiomycota | Agaricomycetes | Polyporales | Phanerochaetaceae | *Phlebiopsis* | 2 |
| Basidiomycota | Agaricomycetes | Agaricales | Psathyrellaceae | *Coprinellus* | 1 |
| Ascomycota | Sordariomycetes | Hypocreales | Nectriaceae | — | 1 |
| Ascomycota | Sordariomycetes | Xylariales | Hypoxylaceae | *Hypoxylon* | 1 |
| Ascomycota | Sordariomycetes | Xylariales | Xylariaceae | *Muscodor* | 1 |
| Basidiomycota | Agaricomycetes | Polyporales | Meruliaceae | *Mycoacia* | 1 |
| Ascomycota | Sordariomycetes | Amphisphaeriales | Amphisphaeiaceae | *Pestalotiopsis* | 1 |
