# Supplementary material for: Double lives: transfer of fungal endophytes from leaves to woody substrates
Source: PeerJ. 2020 Aug 28;8:e9341. doi: 10.7717/peerj.9341 (PMC7457945; doi:10.7717/peerj.9341)
Supplement: Supplemental Information 3 [file peerj-08-9341-s003.docx]

**Table S3: Number of isolates of each genus, and functional guild assigned**

| **Genus** | **LEAF^a^** | | | | | | | | | | | | **Total** | **Functional** | **Guild** |
| --- | --- | --- | --- | --- | --- | --- | --- | --- | --- | --- | --- | --- | --- | --- | --- |
|  | **1** | **2** | **3** | **4** | **5** | **6** | **7** | **8** | **9** | **10** | **11** | **12** | **Isolates** | **Guild** | **Reference** |
| *Penicillium* | 1 | 10 | 9 | 9 | 5 | 9 | 1 | 26 | 26 | 24 | 32 | 27 | 179 | Saprotroph | Nguyen et al., 2016 |
| *Trichoderma* | 2 | 1 |  |  | 16 | 18 | 29 | 14 | 4 | 5 |  |  | 89 | Saprotroph | Nguyen et al., 2016 |
| *Byssochlamys* | 17 | 17 | 12 | 26 | 1 | 1 |  |  |  |  | 1 |  | 75 | Saprotroph | Nguyen et al., 2016 |
| *Neopestalotiopsis* | 16 | 7 | 6 | 15 |  |  |  |  |  |  |  |  | 44 | Path.-Sap. | Maharachchikumbura et al. 2014 |
| *Phanerochaete* | 3 |  | 6 |  |  | 3 | 1 |  | 1 |  |  |  | 14 | Saprotroph | Nguyen et al., 2016 |
| *Diaporthe* | 5 |  |  |  | 5 |  |  |  |  |  |  |  | 10 | Pathotroph | Nguyen et al., 2016 |
| *Fusarium* |  |  |  |  |  |  |  |  | 4 | 4 |  | 1 | 9 | Path.-Sap. | Nguyen et al., 2016 |
| *Aspergillus* |  |  |  |  |  | 2 | 1 | 1 |  | 2 |  |  | 6 | Saprotroph | Nguyen et al., 2016 |
| *Curvularia* |  |  |  |  |  |  |  |  |  | 1 | 5 |  | 6 | Pathotroph | Nguyen et al., 2016 |
| *Peniophora* | 4 | 1 |  |  |  |  |  |  | 1 |  |  |  | 6 | Saprotroph | Nguyen et al., 2016 |
| *Alloconiothyrium* |  |  |  |  | 5 |  |  |  |  |  |  |  | 5 | Unassigned | Verkley et al. 2014 |
| *Kalmusia* |  | 2 |  |  |  |  |  |  | 2 |  |  | 1 | 5 | Pathotroph | Nguyen et al., 2016 |
| *Phlebia* |  |  |  | 3 |  |  |  |  |  | 1 |  |  | 4 | Saprotroph | Nguyen et al., 2016 |
| *Cladophialophora* |  | 3 |  |  |  |  |  |  |  |  |  |  | 3 | Saprotroph | Nguyen et al., 2016 |
| *Xylaria* |  | 3 |  |  |  |  |  |  |  |  |  |  | 3 | Saprotroph | Nguyen et al., 2016 |
| *Colletotrichum* |  |  |  |  |  |  |  |  |  |  |  | 2 | 2 | Pathotroph | Nguyen et al., 2016 |
| *Lophiostoma* |  | 2 |  |  |  |  |  |  |  |  |  |  | 2 | Saprotroph | Nguyen et al., 2016 |
| Dothideomycetes |  |  |  |  | 2 |  |  |  |  |  |  |  | 2 | — | — |
| *Phlebiopsis* |  |  |  | 2 |  |  |  |  |  |  |  |  | 2 | Saprotroph | Nguyen et al., 2016 |
| *Coprinellus* |  |  |  | 1 |  |  |  |  |  |  |  |  | 1 | Saprotroph | Nguyen et al., 2016 |
| Nectriaceae |  | 1 |  |  |  |  |  |  |  |  |  |  | 1 | — | — |
| *Hypoxylon* |  |  |  |  |  |  |  | 1 |  |  |  |  | 1 | Saprotroph | Nguyen et al., 2016 |
| *Muscodor* | 1 |  |  |  |  |  |  |  |  |  |  |  | 1 | Saprotroph | Nguyen et al., 2016 |
| *Mycoacia* |  |  |  |  |  |  |  |  |  |  | 1 |  | 1 | Saprotroph | Nguyen et al., 2016 |
| *Pestalotiopsis* |  | 1 |  |  |  |  |  |  |  |  |  |  | 1 | Pathotroph | Nguyen et al., 2016 |

^a^ Each leaf was inoculated onto two tongue depressors; leaves incubated in the same box are indicated by background color.
