# Supplementary figures and images for: Double lives: transfer of fungal endophytes from leaves to woody substrates
Source: PeerJ. 2020 Aug 28;8:e9341. doi: 10.7717/peerj.9341 (PMC7457945; doi:10.7717/peerj.9341)

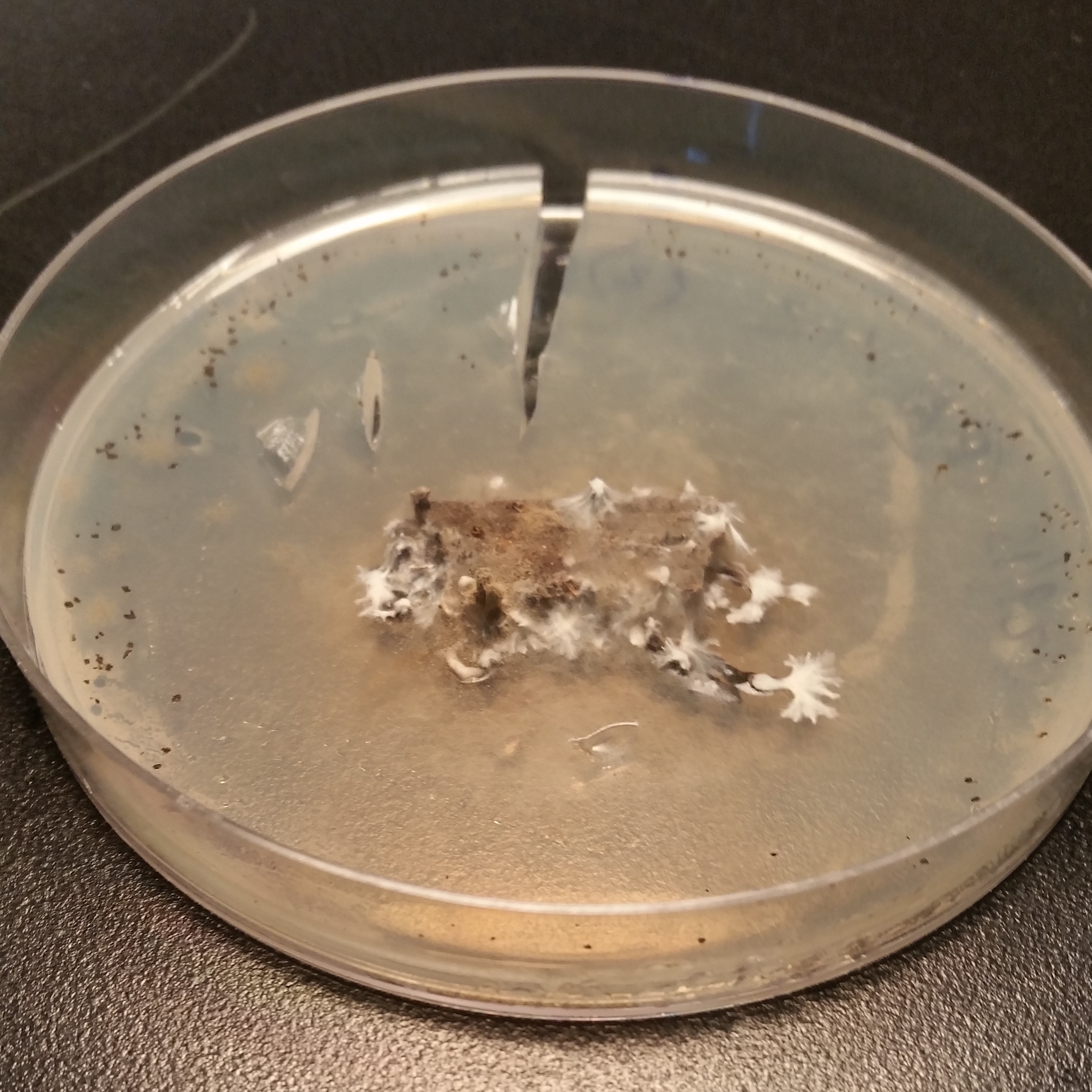

Supplement: Supplemental Information 4 — Growing on wood substrates inoculated by leaf endophytes. [file peerj-08-9341-s004.jpg]
